# ATP13A2 promoted cell stemness, cisplatin resistance, autophagy, and cell progression of bladder cancer

**DOI:** 10.3389/fimmu.2026.1743064

**Published:** 2026-05-07

**Authors:** Chenxi Zhu, Jinjin Lu, Biqiang Zhu, Bing Liu, Guohao Li, Yonglian Guo, Xiaoyan Guo, Zhuo Yin

**Affiliations:** 1 Department of Urology, The Central Hospital of Wuhan, Tongji Medical College, Huazhong University of Science and Technology, Wuhan, China; 2 Key Laboratory for Molecular Diagnosis of Hubei Province, The Central Hospital of Wuhan, Tongji Medical College, Huazhong University of Science and Technology, Wuhan, China; 3 Department of Chinese Medicine, The First Affiliated Hospital of Zhengzhou University, Zhengzhou, Henan, China; 4 Department of Urology, The Second Xiangya Hospital, Central South University, Changsha, Hunan, China

**Keywords:** ATP13A2, autophagy, bladder cancer, cell progression, stemness

## Abstract

**Background:**

ATP13A2 is highly expressed in bladder cancer, but its molecular mechanism remains unclear. This study aims to explore whether ATP13A2 regulates cell stemness, autophagy, cisplatin resistance, and progression in bladder cancer.

**Materials and methods:**

Mouse models and T24 bladder cancer cells were employed. Cells were transfected with ATP13A2-overexpressing vectors or short hairpin RNA (shRNA) targeting ATP13A2 using the Lipofectamine 2000 kit. Tissue morphology was observed by HE staining. ATP13A2 expression was determined by real-time quantitative PCR, western blotting and immunohistochemical (IHC) staining. IHC and immunofluorescence staining were performed to measure LC3 and p62 levels. Cell stemness was assessed using a sphere formation assay. Colony formation, cell counting kit-8 assay, Transwell assays, wound healing test, and flow cytometry were used to evaluate cell proliferation, migration, invasion, and apoptosis. Molecular docking was performed to analyze the interaction between ATP13A2 and the autophagy inhibitor, bafilomycin A1.

**Results:**

ATP13A2 was upregulated in bladder cancer and associated with poor prognosis. Silencing ATP13A2 inhibited cell stemness, cisplatin resistance, and autophagy. Knockdown of ATP13A2 also suppressed cell proliferation, migration, and invasion, while promoting cell apoptosis. Overexpression of ATP13A2 promoted cell stemness, cisplatin resistance, and autophagy. Bafilomycin A1 significantly inhibited cell stemness and reduced drug resistance, suppressing bladder cancer cell progression.

**Conclusion:**

Elevated expression of ATP13A2 promotes cell stemness, cisplatin resistance, autophagy, and cell progression in bladder cancer.

## Highlights

- ATP13A2 was significantly highly expressed in bladder cancer and associated with poor prognosis.
- Silencing ATP13A2 inhibited cell stemness, cisplatin resistance, and autophagy.
- Knockdown of ATP13A2 inhibited the cell progression of bladder cancer.
- Bafilomycin A1 can significantly inhibit cell stemness and weaken drug resistance.
- Knockdown of ATP13A2 inhibited bladder cancer cell growth.

## Introduction

Bladder cancer arises from the mucous membrane of the bladder and ranks as the tenth most common cancer worldwide, with 573,278 cases reported in 2020 (1). Cigarette smoking is the primary risk factor, accounting for approximately 50% of all cases; the incidence is higher in men than in women (2). A combination of cisplatin, 5-FU, leucovorin, and radiotherapy is a viable and promising option for bladder cancer (3, 4). Among patients with invasive bladder cancer who undergo total cystectomy, the five-year survival rate is 60%–70% (5). Drug resistance is a critical factor contributing to therapeutic failure (6). Elucidating the mechanism of cisplatin resistance may help improve survival rates in bladder cancer.

Cancer stem cell progression and tumorigenesis are considered essential factors underlying the high recurrence rate and refractory nature of bladder cancer (7, 8). Due to their quiescence, efficient DNA repair, and resistance to apoptosis, cancer stem cells can evade drug-induced damage and are thus regarded as major contributors to chemoresistance (8). However, the molecular mechanism by which bladder cancer stemness influences cisplatin resistance remains unclear. Therefore, in-depth molecular studies on bladder cancer stemness and chemotherapeutic resistance are of great importance.

ATP13A2 (also known as PARK9) is a lysosomal P5B-type ATPase that has been extensively studied in the context of neurodegenerative disorders (9). It functions as a polyamine and inorganic cation transporter, playing a critical role in maintaining lysosomal homeostasis, autophagic flux, and mitochondrial function (10). Lysosomal dysfunction caused by ATP13A2 mutations leads to impaired autophagy and accumulation of toxic substrates, highlighting its essential role in the autophagy-lysosomal pathway (11, 12). Given that autophagy is increasingly recognized as a key regulator of cancer stemness and chemoresistance, ATP13A2 has recently drawn attention in oncology research. For instance, Alam et al. (13) identified ATP13A2 and its co-expressed proteins as potential biomarkers and therapeutic targets in brain cancer. Another study demonstrated that knockdown of ATP13A2 inhibits tumor formation in colon cancer by interfering with autophagic flux (14). Despite these emerging findings, the role of ATP13A2 in bladder cancer has not been systematically investigated.

According to our preliminary analysis of GEPIA, ATP13A2 is significantly upregulated in bladder cancer tissues compared with normal tissues, and patients with high ATP13A2 expression typically have a poor prognosis. However, it remains unknown whether ATP13A2 regulates bladder cancer progression, stemness, or chemotherapy response, and whether such effects are mediated through autophagy. Given that autophagy activity acts as a molecular switch for stem cell differentiation and stemness maintenance (15), we hypothesized that ATP13A2 may influence cisplatin resistance and cell stemness in bladder cancer via the autophagic pathway.

In this study, we first verified the role of ATP13A2 in regulating bladder cancer stemness, autophagy, and cisplatin resistance. Then, we investigated the molecular mechanism of ATP13A2 in affecting bladder cancer cell progression. This study may provide new insights into the role of ATP13A2 in bladder cancer and identify a potential therapeutic target for reducing chemoresistance.

## Materials and methods

### Cell culture and interventions

Human bladder cancer T24 cells were obtained from the American Type Culture Collection (Manassas, VA, USA) and cultured in high-glucose DMEM supplemented with 10% fetal bovine serum (FBS, Gibco, USA) and 1% penicillin-streptomycin at 37°C in 5% CO_2_.

T24 cells at 80%–90% confluence were transfected with lentiviral particles overexpressing ATP13A2 (), short hairpin RNA (shRNA) targeting ATP13A2 (sh-ATP13A2), and their corresponding negative controls using the Lipofectamine 3000 kit (Thermo Fisher Scientific, Wlatham, MA, USA). According to the manufacturer’s instructions, cells were seeded at a density of approximately 2 × 10^5^ cells/well in 6-well plates and transfected with lentiviral particles at a multiplicity of infection (MOI) of 20. After 6 h incubation, the medium was replaced with fresh complete medium, and cells were cultured for an additional 48 h before subsequent experiments. Transfection efficiency was verified by quantitative polymerase chain reaction (qPCR). Where indicated, cells were treated with 10 nM bafilomycin A1, an autophagy inhibitor.

### Animals experiment

Thirty six-week-old male BALB/c mice were obtained from the Animal Center of the Chinese Academy of Sciences (Shanghai, China). Animals were housed in polypropylene cages under a 12 h:12h dark/light cycle and provided with food and water *ad libitum*. T24 cells transfected with sh-NC (sh-NC group, n = 6) or sh-ATP13A2 (sh-ATP13A2 group, n = 6) were injected into the mice. Additionally, six-week-old male C57BL/6 mice were used to establish an MB49 syngeneic bladder cancer model. MB49 cells (1 × 10^6^ in 100 μL PBS) were subcutaneously injected into the right flank. Mice were randomly assigned to sh-NC (n=6) and sh-ATP13A2 (n=6) groups. Tumor size was measured every 3 days using a digital caliper. At the end of the study, animals were anesthetized and sacrificed. Tumor volume was calculated as length × (width)^2^/2. Tumor weight was also measured.

### H&E, immunohistochemical, and immunofluorescence staining

Tumor tissues were perfused with heparinized physiological saline and 4% paraformaldehyde, then embedded in paraffin and sectioned coronally at 4 μm. Sections were stained using an H&E kit (Solarbio, China).

For IHC analysis, sections were incubated with primary antibodies against ATP13A2, LC3, p62, PD-L1 and CD8 at 4 °C for 12 h, followed by incubation with HRP-labeled goat anti-rabbit IgG secondary antibody at room temperature for 40 min.

Immunofluorescence was used to analyze LC3 and p62 levels in cells. Cells were washed three times with PBS, fixed with 4% paraformaldehyde for 15 min, permeabilized with 0.1% Triton X-100 for 10 min, and blocked with 5% BSA,. Subsequently, cells were incubated with anti-LC3 and anti-p62 antibodies at 4 °C for 12 h, followed by HRP-labeled goat anti-rabbit IgG secondary antibody at room temperature for 40 min.

### TUNEL staining

TUNEL assay was conducted under the *In-Situ* Cell Death Detection Kit (Roche, Germany) following the manufacturer’s guidelines. Tissue sections were incubated with an enzyme solution tagged with red fluorescence and then analyzed under a fluorescence microscope (DM2500; Leica Microsystems, Germany). Five fields were selected from each section. Images were merged after completion of the experiment.

### Real-time qPCR

Total RNA was extracted using TRIzol reagent (Invitrogen, USA). Primers for ATP13A2 and β-actin were designed using Primer Premier 5 and synthesized by Sangon (Shanghai, China). An one-step amplification kit (#Q221-01) was employed. The products were analyzed on an ABI 7900 system (Foster City, CA, USA). Expression levels were calculated using the 2^-ΔΔCt^ method.

### Western blotting

Protein was extracted from samples using RIPA lysis buffer (#R0278, Sigma). Western blot analysis was performed using standard SDS-PAGE method. Briefly, proteins were separated at 100 V, processed in concentration gel at 120 V, and blocked overnight. The gels were incubated with primary antibodies against ATP13A2 (19141-AP), Bax (ab32503), Bcl-2 (ab59348), Caspase-3 (ab13847), phosphorylated mTOR (p-mTOR; AP0094), mTOR (A2445), AMPK (A1229), phosphorylated AMPK (p-AMPK; 80209-6-RR), TFEB (A21657), PD-L1 (A26086), MHC-I (A8754), LC3-I/LC3-II (A5618), p62 (A7758), and β-actin (ab227387). Samples were incubated with HRP-labeled goat anti-rabbit IgG secondary antibody for 1 h at room temperature. PVDF membranes were exposed to electrogenerated chemiluminescence (ECL808-25, Biomiga, San Diego, CA, USA) for 1 min.

### Cell sphere formation assay

T24 cells and co-cultured cells were seeded in serum-free DMEM/F12 (Gibco) supplemented with 20 ng/ml EGF (Gibco), 2% B27 (Gibco), and 20 ng/mL basic FGF (Gibco). The number of spheres was counted using a microscope (Olympus, Tokyo, Japan).

### Colony formation

Colony formation assay was performed to evaluate cell proliferation. Approximately 800 cells from each group were cultured. Ten days later, cells were trypsinized, fixed, stained with 0.1% crystal violet (Sigma-Aldrich; Merck KGaA), and examined under a light microscope.

### Flow cytometry

After treatment with cisplatin, transfected cells were analyzed using the Annexin V FITC/PI Apoptosis Detection kit (Sigma-Aldrich). Approximately 3 × 10^4^ cells were suspended in binding buffer and incubated with annexin V-FITC and PI stains. Cells were analyzed by flow cytometry (Agilent, USA) using winMDI 2.9 software.

### Cell counting kit-8 assay

Cells were treated with graded concentrations of cisplatin (0, 10, 50, 100, and 200 μg/mL), vector, overexpressed ATP13A2, or sh-ATP13A2 for 0, 12, 24, 48, and 72 h. They were incubated with CCK-8 reagents in 5% CO_2_ at 37 °C for 4 h. Absorbance was measured at 450 nm using a microplate reader.

### Wound healing assay

Cell migration was also assessed using a wound healing assay. Stably transfected cells were seeded into 6-well plates, cultured for over 12h, and then scratched with a sterile plastic pipette tip. Cells were washed and cultured in a medium containing 1% FBS for two days.

### Transwell assays

Cell migration and invasion abilities were measured using Transwell assays. For invasion analysis, the apical chamber was seeded with approximately 1.0×10^5^ cells in each well in serum-free DMEM and incubated for 6 h at 37°C. The basolateral chamber was filled with a medium containing 10% FBS. Transwell membranes were washed, fixed with glutaraldehyde, and stained with 0.1% crystal violet. Cells were counted in five fields under a 400× microscope.

### Bioinformatics analysis

The GEPIA2 database was used to predict ATP13A2 expression in bladder cancer. Public databases GPL6102 and GSE13507 were employed. Differentially expressed genes (DEGs) were screened with a threshold of *P* < 0.05 and |log_2_fold change (FC)| >1. Kyoto Encyclopedia of Genes and Genomes (KEGG) analysis was performed using the DAVID database. Additionally, ATP13A2 expression comparison, survival analysis, and ROC diagnostic evaluation were performed using the TCGA-BLCA dataset to further support clinical relevance.

RNA-seq data and clinical information for bladder cancer patients were obtained from the TCGA-BLCA database. StromalScore, ImmuneScore, and ESTIMATEScore were calculated using the “estimate” R package. Relative infiltration levels of immune cells were assessed using the ssGSEA algorithm in the “GSVA” R package. Spearman correlation analysis was performed to evaluate the associations between ATP13A2 expression and immune cell infiltration or immune-related gene expression.

### Molecular docking

The protein crystal structure of human ATP13A2 (PDB ID: 7M5X) was obtained from the UniProt database. No ligand for this protein has been previously reported. The sitemap tool in Schrödinger was used to predict binding pockets on the protein surface. The top-ranked pocket was selected for high-throughput virtual screening. High-speed docking was performed using Schrödinger’s Glide tool with precision set to HTVS and two poses per ligand. The screened library included FDA-approved drugs (3,158 compounds) and an Anti-Cancer Compound Library (7,324 compounds). Results were sorted by docking score in descending order, and the top 1% of compounds were selected. Precision was then adjusted to XP for fine docking of these compounds. Possible ligands of ATP13A2 were identified through this screening process. Molecular dynamics (MD) simulations of the ATP13A2/bafilomycin A1 complex were performed using the Desmond tool to assess binding stability. MD simulations were conducted using an orthorhombic box under NPT conditions at 300 K, 1.013 bar, with 0.15 M NaCl for 1000 ns.

### Cellular thermal shift assay

T24 bladder cancer cells were cultured to approximately 80% confluence and divided into control and bafilomycin A1–treated groups. Cells were treated with bafilomycin A1 (10 nM) for 2 h, collected, resuspended in PBS, and subjected to heat treatment at 46°C, 50°C, 54°C, or 58°C for 3 min, followed by immediate cooling on ice. Cells were then lysed with RIPA buffer and centrifuged. The soluble fractions were analyzed by Western blotting to assess ATP13A2 protein levels. Thermal stability was evaluated by comparing ATP13A2 protein retention across temperature points.

### Statistical analysis

Unpaired student’s *t*-test and one-way ANOVA analyses were performed as appropriate. Graphs were generated using GraphPad Prism 10 (San Diego, CA, USA). Data are presented as mean ± standard deviation (SD). All experiments were performed at least three independent times. Comparisons between two groups were conducted using unpaired Student’s t-test, while comparisons among multiple groups were analyzed using one-way ANOVA followed by Tukey’s *post hoc* test. A *P* < 0.05 was considered as significantly different.

## Results

### ATP13A2 is upregulated in bladder cancer and is associated with poor prognosis

**Figure 1:**
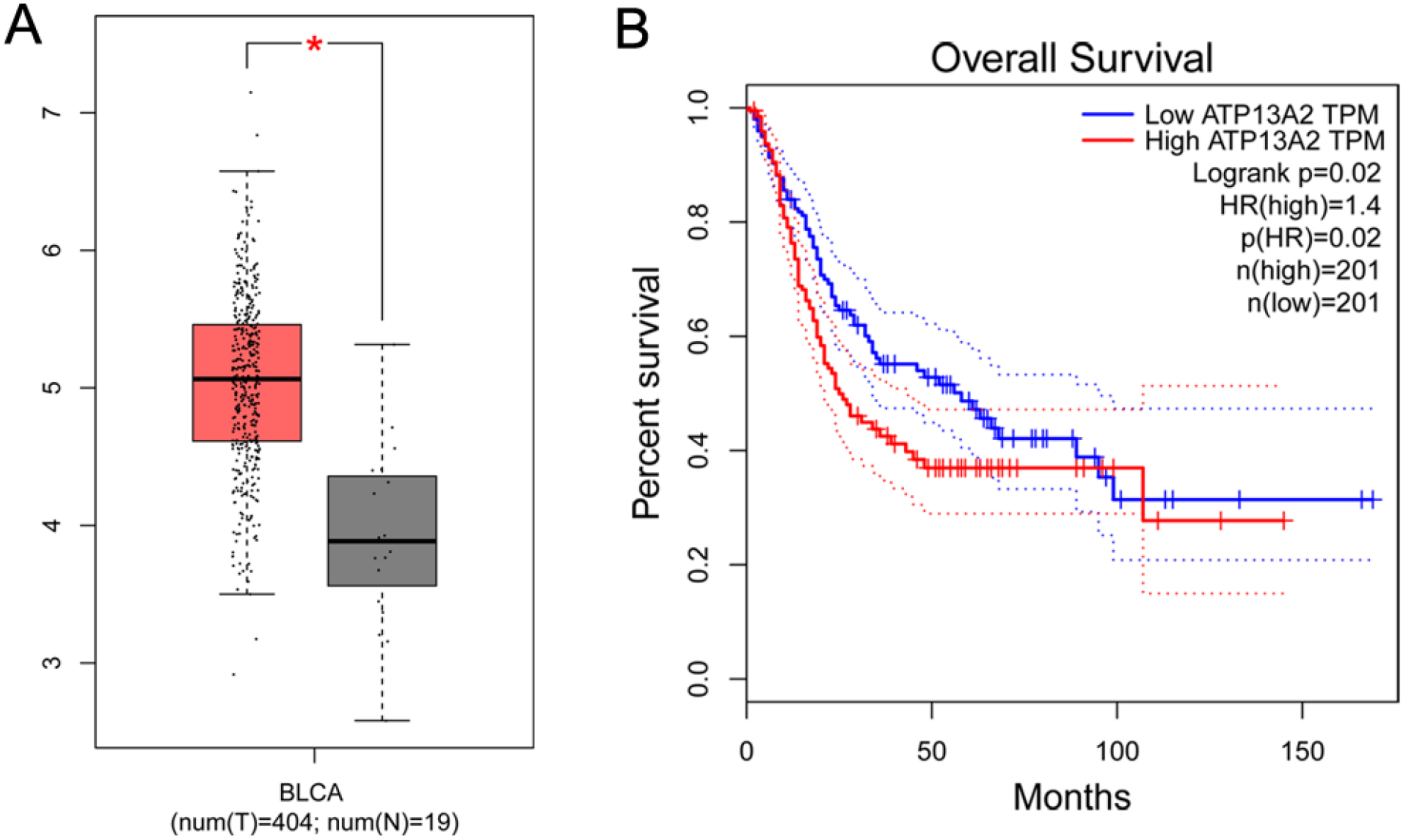
ATP13A2 was significantly highly expressed in bladder cancer and associated with poor prognosis. **(A)** The GEPIA database was used to analyze the expression level of ATP13A2 in bladder cancer and normal tissues. Data are presented as mean ± SD, and statistical significance was determined by Student’s t-test. **(B)** The GEPIA database was used to evaluate the overall survival of bladder cancer patients with different ATP13A2 expression levels. Kaplan–Meier survival curves and the log-rank test were applied to assess statistical significance.

As detected by GEPIA, ATP13A2 was upregulated in bladder cancer (*P* < 0.05, **Figure 1A**). and its higher expression was significantly associated with poorer prognosis (*P* = 0.02, **Figure 1B**). Consistently, analysis of the TCGA-BLCA cohort further confirmed the clinical relevance of ATP13A2, showing markedly elevated expression in tumor tissues compared with normal controls (*P* < 0.001). Patients with high ATP13A2 expression exhibited significantly reduced overall survival (*P* = 0.012). Moreover, ROC analysis indicated strong diagnostic potential, with an AUC of 0.873 (95% CI: 0.806–0.941) (**Supplementary Figure 1**).

### Knockdown of ATP13A2 inhibited the cell progression of bladder cancer

**Figure 2:**
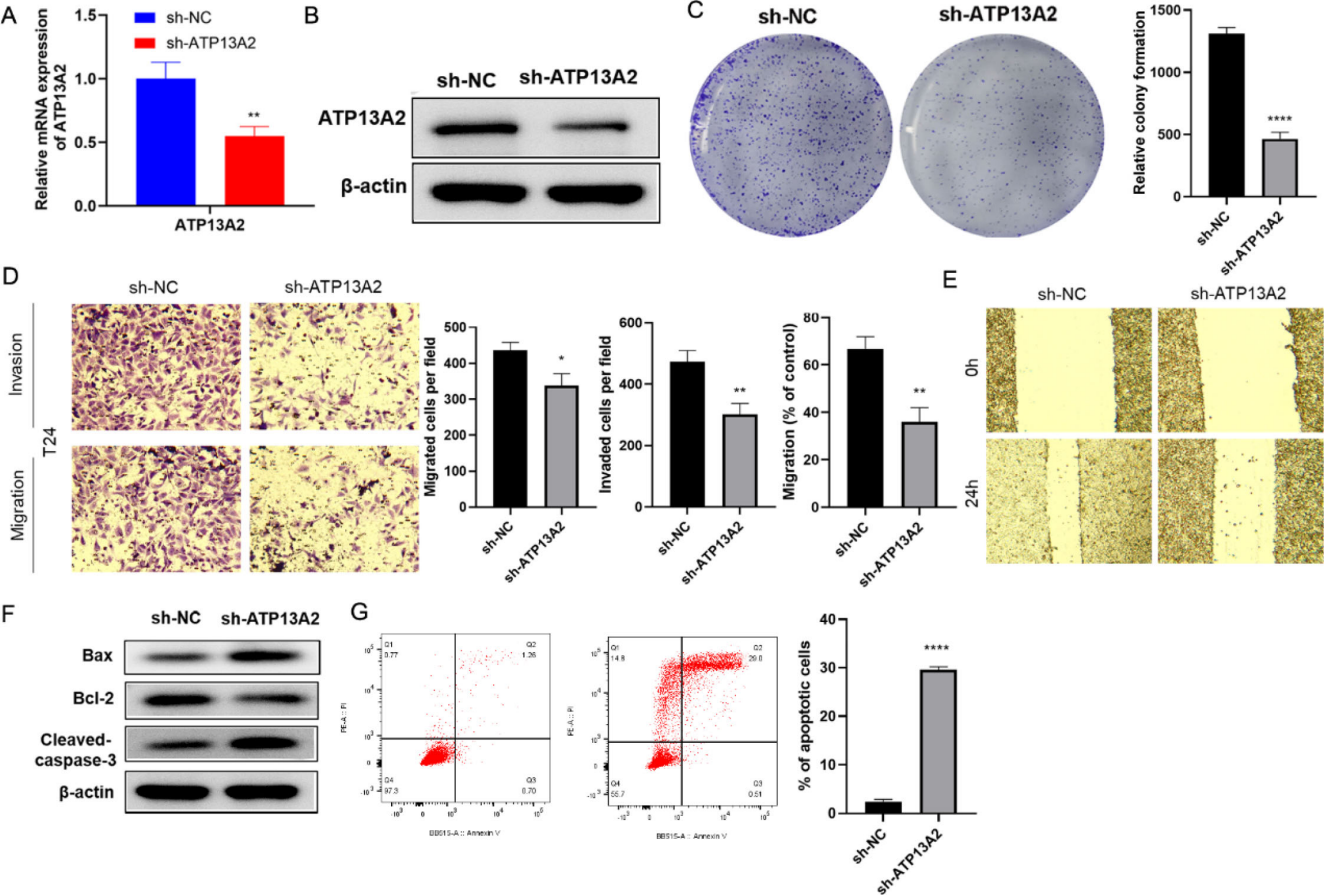
Knockdown of ATP13A2 inhibited cell activity, migration, and invasion and promoted cell apoptosis in bladder cancer. **(A)** QPCR analysis of ATP13A2 mRNA expression in T24 cells transfected with sh-NC or sh-ATP13A2. **(B)** WB analysis of ATP13A2 protein levels in transfected cells. **(C)** Colony formation assay to evaluate cell proliferation. **(D)** Transwell assays to assess cell migration and invasion in transfected cells. **(E)** Wound-healing assay to measure cell migration rate. **(F)** WB analysis of apoptosis-related proteins (e.g., Cleaved caspase-3, Bax, Bcl-2). **(G)** Flow cytometry to analyze apoptosis ratio in transfected cells. N = 3, data are presented as mean ± SD. Statistical significance between the two groups was determined by unpaired Student’s t-test. *P < 0.05, **P < 0.01, ****P < 0.0001.

To further verify the function of ATP13A2 in regulating bladder cancer cell progression, T24 cells were transfected with sh-ATP13A2 or ATP13A2-overexpressing vectors. ATP13A2 expression was assessed in the sh-NC and sh-ATP13A2 groups to confirm successful transfection. As detected by qPCR and Western blotting, ATP13A2 expression was significantly decreased in the sh-ATP13A2 group compared with the sh-NC group (*P* < 0.001, **Figures 2A, B**), demonstrating successful transfection. Subsequently, the role of ATP13A2 in regulating bladder cancer cell activities was measured. Colony formation assays revealed that cell proliferation was notably inhibited by sh-ATP13A2 compared with sh-NC (**Figure 2C**). Knockdown of ATP13A2 also reduced cell migration and invasion abilities (*P* < 0.001, **Figure 2D**). We further assessed cell migration using a wound healing assay. Cells in the sh-ATP13A2 group exhibited a significant slower migration rate compared with the sh-NC group (*P* < 0.001, **Figure 2E**). Analysis of apoptosis biomarkers showed that expression levels of Bax and caspase-3 were increased, while Bcl2 was decreased in the sh-ATP13A2 group compared with the sh-NC group (**Figure 2F**). The apoptotic cell ratio was significantly higher in the sh-ATP13A2 group than in the sh-NC group (*P* < 0.05, *P* < 0.001; **Figure 2G**). Taken together, these results indicate that knockdown of ATP13A2 inhibits cell migration and invasion, while promoting cell apoptosis in bladder cancer.

### Bladder cancer progression may be associated with autophagy

**Figure 3:**
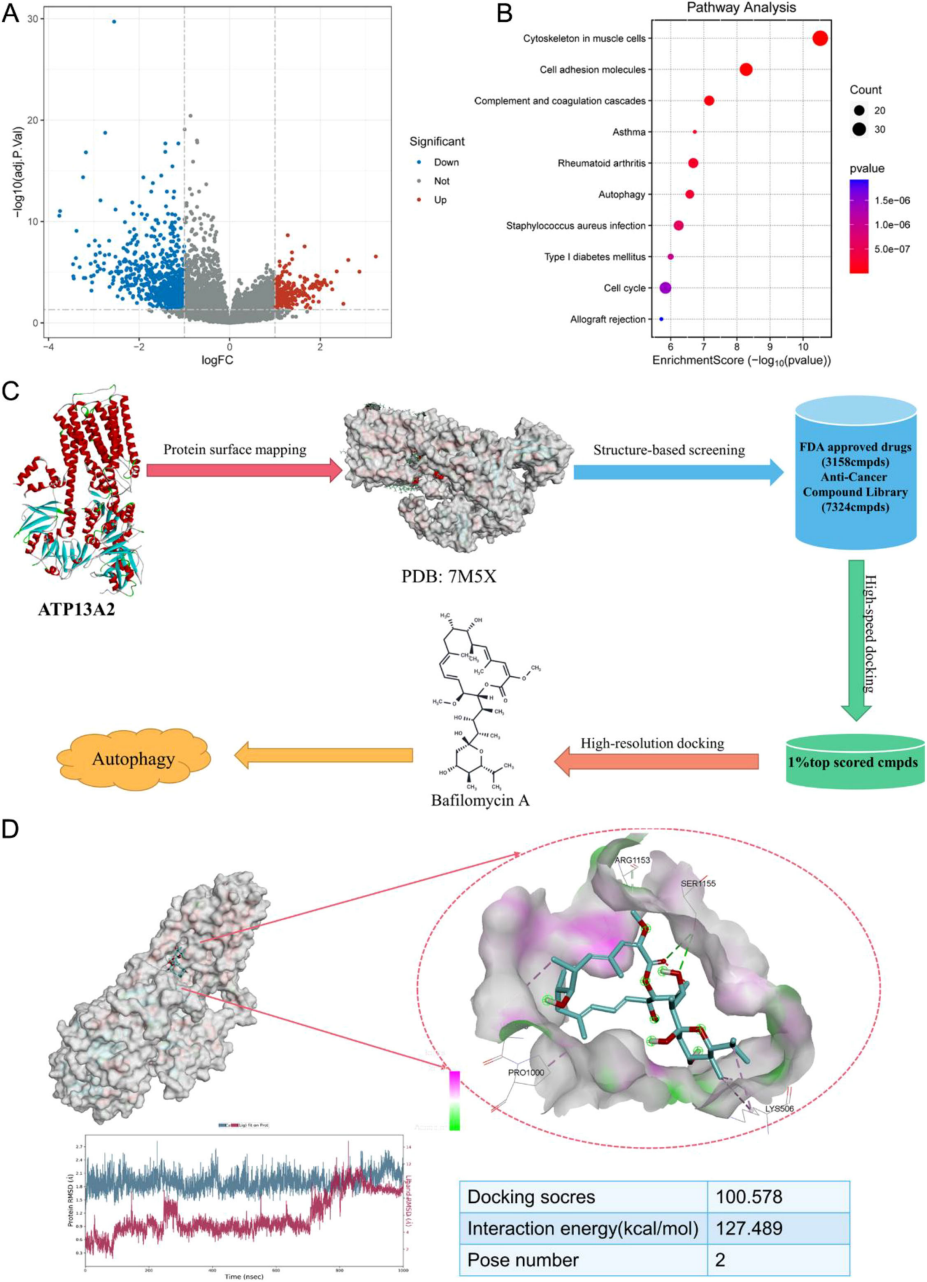
Bladder cancer progression might be associated with autophagy. **(A)** The volcano plot showing DEGs, differentially expressed genes in bladder cancer. **(B)** KEGG pathway analysis of DEGs to identify significantly enriched pathways. **(C)** RMSD curve analyzing the stability of the ATP13A2–Bafilomycin A1 complex during molecular dynamics simulation. **(D)** Docking score of bafilomycin A1 binding to ATP13A2.

Two public databases, GPL6102 and GSE13507, were employed. DEGs were identified, and KEGG analysis demonstrated that these genes were mainly enriched in autophagy-related pathways (**Figures 3A, B**). According to the RMSD curve, the binding of the ATP13A2/bafilomycin A1 complex was stable within 1000 ns. The docking score of bafilomycin A1 was 100.578, and the interaction energy was 127.489 kcal/mol. Bafilomycin A1 interacted with ATP13A2 through hydrogen bonds, specifically via Ser1155, forming a complex that served as an anchor for stable binding. Additional interactions were observed among Pro1000, Lys506, Arg1153, and ATP13A2 (**Figures 3C, D**). To further validate this predicted interaction, CETSA was performed in T24 cells. The results showed that ATP13A2 thermal stability gradually decreased with increasing temperature in the control group, whereas bafilomycin A1 treatment markedly enhanced ATP13A2 stability, with higher protein retention at each temperature point compared with the control, supporting that bafilomycin A1 binds to ATP13A2 and increases its protein stability (**Supplement Figure 2**).

### Knockdown of ATP13A2 inhibited cell stemness, cisplatin resistance, and autophagy

**Figure 4:**
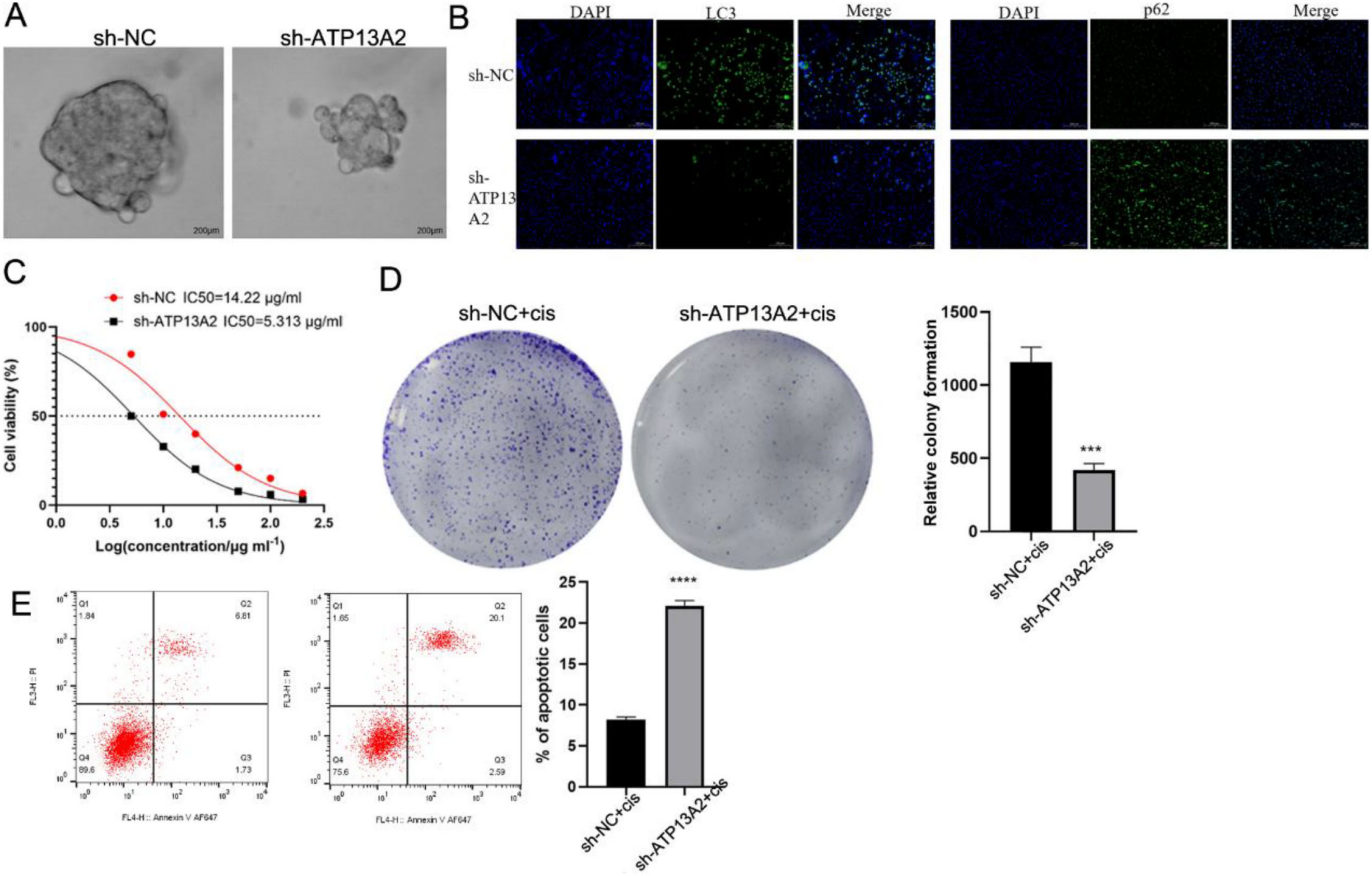
Knockdown of ATP13A2 inhibited autophagy, decreased stemness, and drug resistance. **(A)** Tumor spheroid growth was performed to assess the cell stemness in T24 cells transfected with sh-NC or sh-ATP13A2. **(B)** The autophagy biomarkers of LC3 and p62 were detected by immunofluorescence in transfected cells. **(C)** CCK-8 assay determined the cell activity under graded cisplatin treatments (0, 10, 50, 100, and 200 μg/mL). **(D)** Colony formation was conducted to detect the cell activity under cisplatin treatment at an IC25 concentration in transfected cells. **(E)** Flow cytometry assessed the cell apoptosis under cisplatin treatment at an IC_25_ concentration in transfected cells. N = 3, data are presented as mean ± SD. Statistical significance between the two groups was determined by unpaired student’s t-test. ***P < 0.001, ****P < 0.0001.

Tumor spheroid growth was remarkably reduced in T24 cells transfected with sh-ATP13A2, indicating that sh-ATP13A2 inhibits bladder cancer cell stemness (**Figure 4A**). We assessed autophagy markers in the sh-NC and sh-ATP13A2 groups. Cells transfected with sh-ATP13A2 exhibited lower LC3 levels, higher p62 levels, an increased p-mTOR/mTOR ratio, a decreased p-AMPK/AMPK ratio, and reduced TFEB expression compared with the sh-NC group (**Figure 4B**; **Supplementary Figure 3**), suggesting that ATP13A2 knockdown may suppress autophagy through the AMPK/mTOR/TFEB pathway. To further investigate the role of the AMPK/mTOR/TFEB pathway, sh-ATP13A2 cells were treated with the AMPK activator, AICAR. Western blot analysis showed that AICAR partially restored p-AMPK/AMPK, p-mTOR/mTOR, TFEB, LC3-II, and p62 levels compared with sh-ATP13A2 alone (**Supplementary Figure 4A**). Consistently, sphere formation was partially rescued by AICAR treatment (**Supplementary Figure 4B**), indicating that AMPK activation can partially reverse the effects of ATP13A2 knockdown on autophagy and cell stemness. Then, the cells were treated with graded (0, 10, 50, 100, and 200μg/mL) cisplatin, and the cell activity was evaluated using a CCK-8 assay. Compared with the sh-NC group, the IC50 was significantly decreased in the sh-ATP13A2 group (*P* < 0.001, **Figure 4C**). Colony formation on detecting the cell activity showed that cells transfected with sh-ATP13A2+cisplatin exhibited a lower cell activity than sh-NC+cisplatin (**Figure 4D**). Cells were cultured with an IC_25_ concentration of cisplatin, and apoptosis was detected after 48 h. We found that sh-ATP13A2 combined with cisplatin enhanced cell apoptosis (**Figure 4E**). Therefore, sh-ATP13A2 inhibited cell stemness, cisplatin resistance, and autophagy, and these effects could be partially rescued by AMPK activation via AICAR.

### Overexpressed ATP13A2 promoted cell stemness, cisplatin resistance, and autophagy

**Figure 5:**
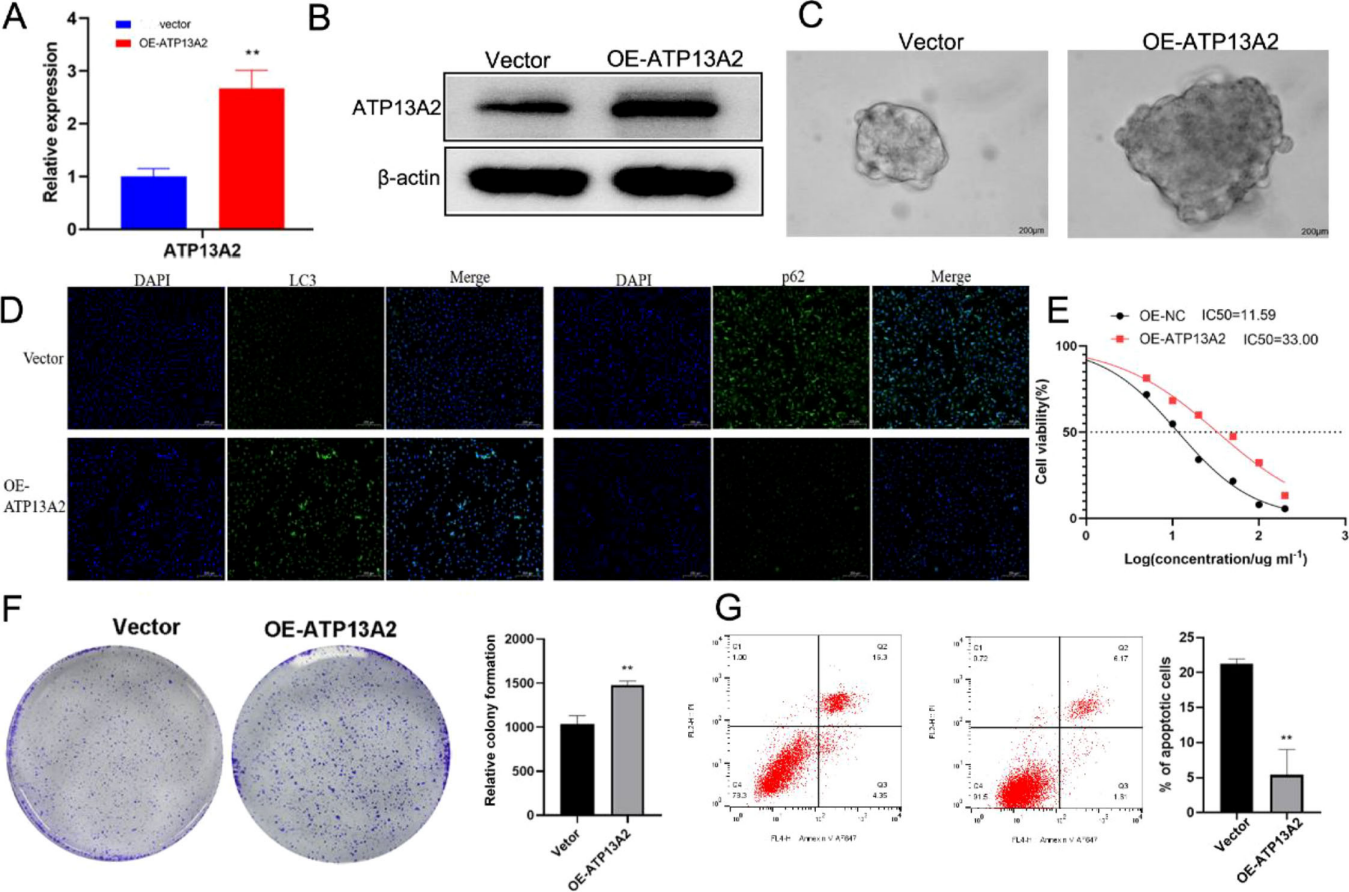
Overexpressed ATP13A2 promoted cell stemness, cisplatin resistance, and autophagy. **(A)** QPCR analysis determined the mRNA expression level of ATP13A2 in T24 cells transfected with vector or ATP13A2 overexpression plasmid. **(B)** WB analysis evaluated the protein expression level of ATP13A2 in transfected cells. **(C)** Tumor spheroid growth was performed to assess the cell stemness in transfected cells. **(D)** The autophagy biomarkers of LC3 and p62 were detected by immunofluorescence in transfected cells. **(E)** CCK-8 assay determined the cell activity under graded cisplatin treatments (0, 10, 50, 100, and 200μg/mL). **(F)** Colony formation was conducted to detect the cell activity under cisplatin treatment at an IC_25_ concentration in transfected cells. **(G)** Flow cytometry assessed the cell apoptosis under cisplatin treatment at an IC_25_ concentration in transfected cells. N = 3, data are presented as mean ± SD. Statistical significance between the two groups was determined by unpaired student’s t-test. **P < 0.01.

In overexpression experiments, the role of ATP13A2 in cell stemness, cisplatin resistance, autophagy, and cell progression was determined. As assessed, ATP13A2 was significantly highly expressed in the OE-ATP13A2 group compared with the vector control group (*P* < 0.001, **Figures 5A, B**). Tumor spheroid growth was significantly increased in T24 cells transfected with overexpressed ATP13A2, indicating that ATP13A2 can promote bladder cancer cell stemness (**Figure 5C**). Higher levels of LC3, lower levels of p62, a decreased p-mTOR/mTOR ratio, an increased p-AMPK/AMPK ratio, and elevated TFEB expression were observed in cells transfected with OE-ATP13A2 (**Figure 5D**; **Supplementary Figure 5**), demonstrating that ATP13A2 promotes autophagy. Graded cisplatin treatments were used to assess drug resistance in T24 cells. The IC_50_ was significantly increased in the OE-ATP13A2 group compared to the vector group (*P* < 0.001, **Figure 5E**), showing enhanced cell viability by overexpressed ATP13A2. Cell proliferation under cisplatin treatment improved remarkably, and cell apoptosis was significantly inhibited by ATP13A2 (*P* < 0.001, **Figures 5F, G**). We conclude that overexpressed ATP13A2 promotes cell stemness, cisplatin resistance, and autophagy.

### ATP13A2 is associated with an immunosuppressive tumor immune microenvironment

To investigate the relationship between ATP13A2 and the tumor immune microenvironment in bladder cancer, we performed immune infiltration analyses based on TCGA-BLCA data. ATP13A2 expression was significantly negatively correlated with StromalScore and ESTIMATEScore (StromalScore: R = −0.132, *P* < 0.01; ESTIMATEScore: R = −0.111, *P* < 0.05), suggesting that tumors with high ATP13A2 expression may have reduced stromal and immune content (**Supplementary** **Figure 6A**). ssGSEA analysis showed that ATP13A2 was positively correlated with Th2 and γδ T cells, but negatively correlated with B cells, CD8^+^ T cells, and pDCs (**Supplementary Figures 6B, C**), and was significantly associated with multiple immune-related genes, including CD274, HLA-A/B, CD79A, MS4A1, and CD4 (**Supplementary Figure 6D**), indicating a potential role in shaping an immunosuppressive tumor microenvironment.

**Figure 6:**
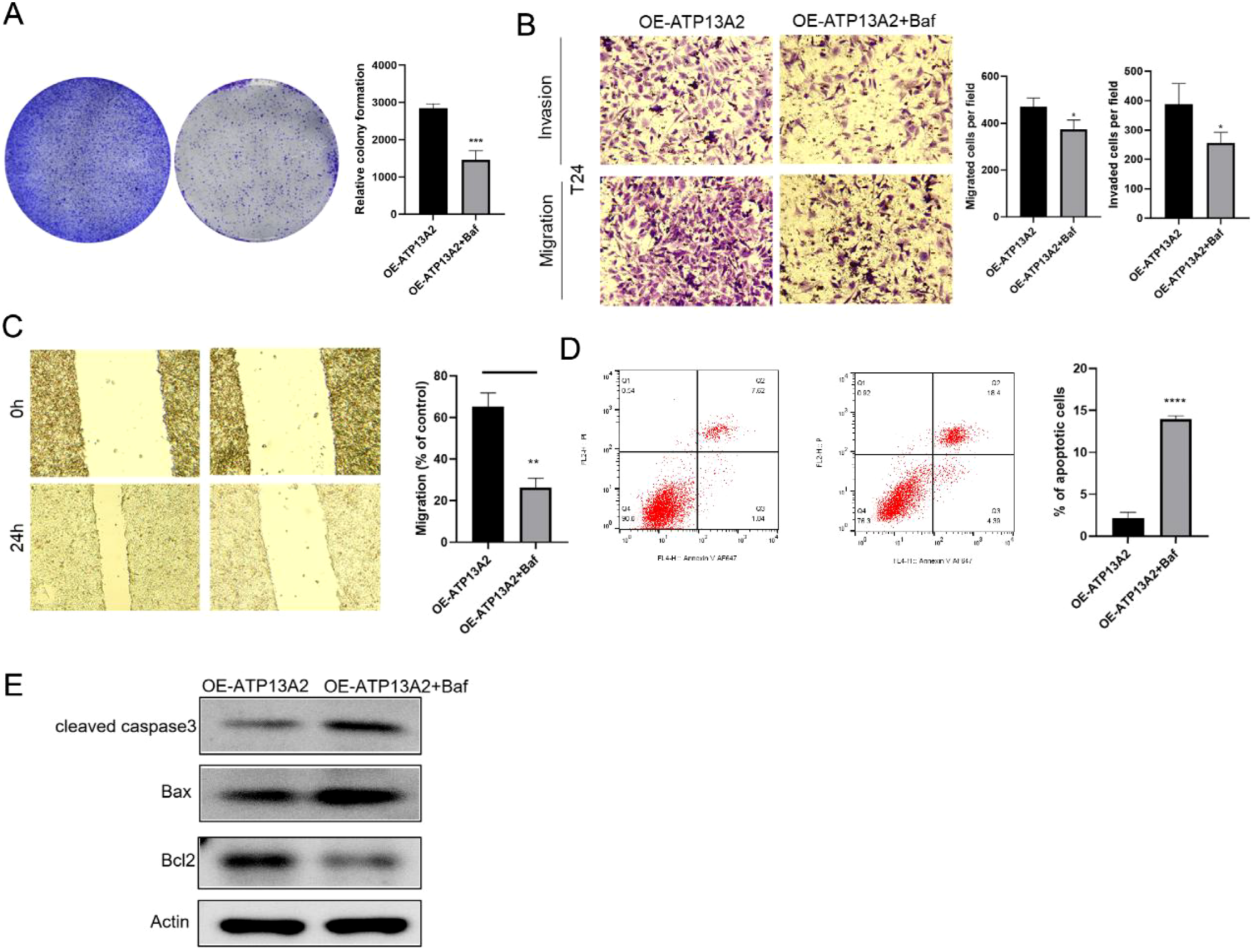
Autophagy inhibitors inhibited cell activity, migration, and invasion and promoted apoptosis of bladder cancer cells. **(A)** Colony formation was performed to evaluate cell proliferation in T24 cells treated with bafilomycin A1. **(B)** Transwell assays assessed the cell migration and invasion in treated cells. **(C)** Wound-healing assay measured the cell migration rate in treated cells. **(D)** The cell apoptosis ratio was observed in treated cells by flow cytometry. **(E)** WB analysis was performed to assess the expression levels of apoptosis-related proteins in cells treated with bafilomycin A1. N = 3, data are presented as mean ± SD. Statistical significance between the two groups was determined by unpaired student’s t-test. *P < 0.05, **P < 0.01, ***P < 0.001, ****P < 0.0001.

*In vitro* experiments in T24 bladder cancer cells further validated these findings. Western blot analysis showed that ATP13A2 knockdown decreased PD-L1 expression and increased MHC-I (HLA-A/B) levels, whereas ATP13A2 overexpression had the opposite effect (**Supplementary Figure 6E**). These results are consistent with the bioinformatic analyses, suggesting that ATP13A2 may contribute to the formation of an immunosuppressive tumor microenvironment by modulating immune checkpoint and antigen presentation molecules.

### Autophagy inhibitors significantly inhibited the cell progression of bladder cancer

Subsequently, the function of the autophagy inhibitor, bafilomycin A1 in regulating bladder cancer was determined. Colony formation assays revealed that cell proliferation was notably inhibited in the OE-ATP13A2 + Baf group compared with the OE-ATP13A2 group (**Figure 6A**). The autophagy inhibitor notably reduced the number of migrating and invading cells (*P* < 0.001, **Figure 6B**). We further assessed cell migration using a wound healing assay. Cells in OE-ATP13A2 + Baf group exhibited a significant slow migration rate compared with the OE-ATP13A2 group (*P* < 0.001, **Figure 6C**). Compared with the apoptosis cell ratio in the OE-ATP13A2 group, it was significantly increased in the OE-ATP13A2+Baf group (*P* < 0.05, *P* < 0.001; **Figure 6D**). The apoptosis-related proteins of Bax and caspase-3 were increased, and Bcl-2 was decreased in the OE-ATP13A2+Baf group when compared with the OE-ATP13A2 group (**Figure 6E**). Taken together, the autophagy inhibitor inhibits cell activity, migration, and invasion and promotes apoptosis of bladder cancer cells.

### Autophagy inhibitor bafilomycin A1 can significantly inhibit cell stemness and weaken drug resistance

**Figure 7:**
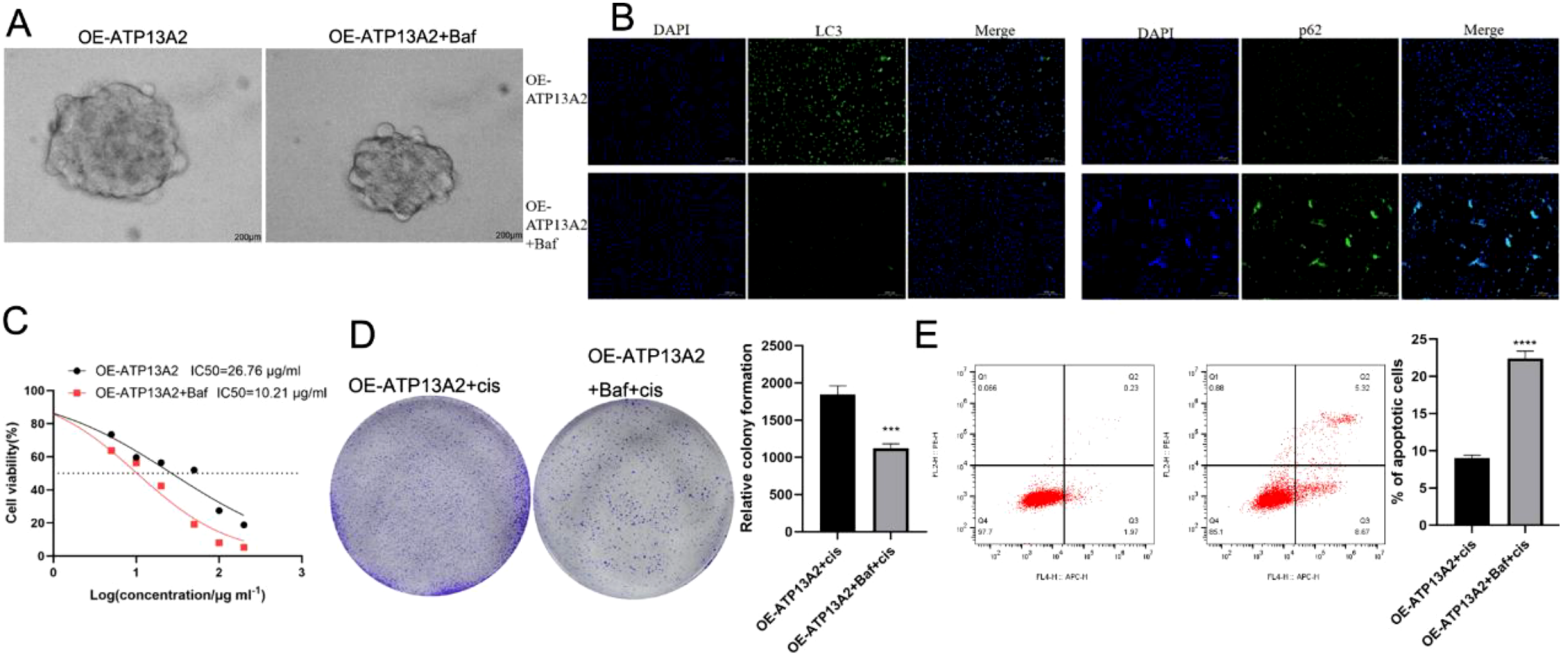
Autophagy inhibitor can significantly inhibit cell stemness and weaken drug resistance. **(A)** Tumor spheroid growth was performed to assess the cell stemness in T24 cells treated with bafilomycin A1. **(B)** The autophagy biomarkers of LC3 and p62 were detected by immunofluorescence (DAPI was used for nuclear staining) in treated cells. **(C)** CCK-8 assay determined the cell activity under graded cisplatin treatments (0, 10, 50, 100, and 200μg/mL). **(D)** Colony formation was conducted to detect the cell activity under cisplatin treatment at an IC25 concentration in treated cells. **(E)** Flow cytometry assessed the cell apoptosis under cisplatin treatment at an IC_25_ concentration in treated cells. N = 3, data are presented as mean ± SD. Statistical significance between the two groups was determined by unpaired student’s t-test. ***P < 0.001, ****P < 0.0001.

We further investigated the autophagy function regulated by ATP13A2 by adding the autophagy inhibitor bafilomycin A1 to OE-ATP13A2 transfections. After treatment with bafilomycin A1, tumor spheroid growth ability was significantly inhibited (**Figure 7A**), indicating an inhibitory role of autophagy inhibitor on cancer cell stemness. LC3 levels were notably decreased, and p62 levels were remarkably elevated in the OE-ATP13A2 + Baf group (*P* < 0.001, **Figure 7B**), revealing the success of autophagy inhibitor treatment. Moreover, inhibited autophagy also influenced cisplatin resistance. The IC50 value was significantly decreased in the OE-ATP13A2+Baf group compared to the OE-ATP13A2 group (*P* < 0.001, **Figure 7C**). After co-culture with cisplatin, the cell activity and apoptosis were measured. bafilomycin A1 significantly reduced cell proliferation and enhanced cell apoptosis (*P* < 0.001, **Figures 7D, E**). These results showed that the autophagy inhibitor bafilomycin A1 can dramatically inhibit cell stemness and weaken drug resistance.

### ATP13A2 promoted bladder cancer cell growth

**Figure 8:**
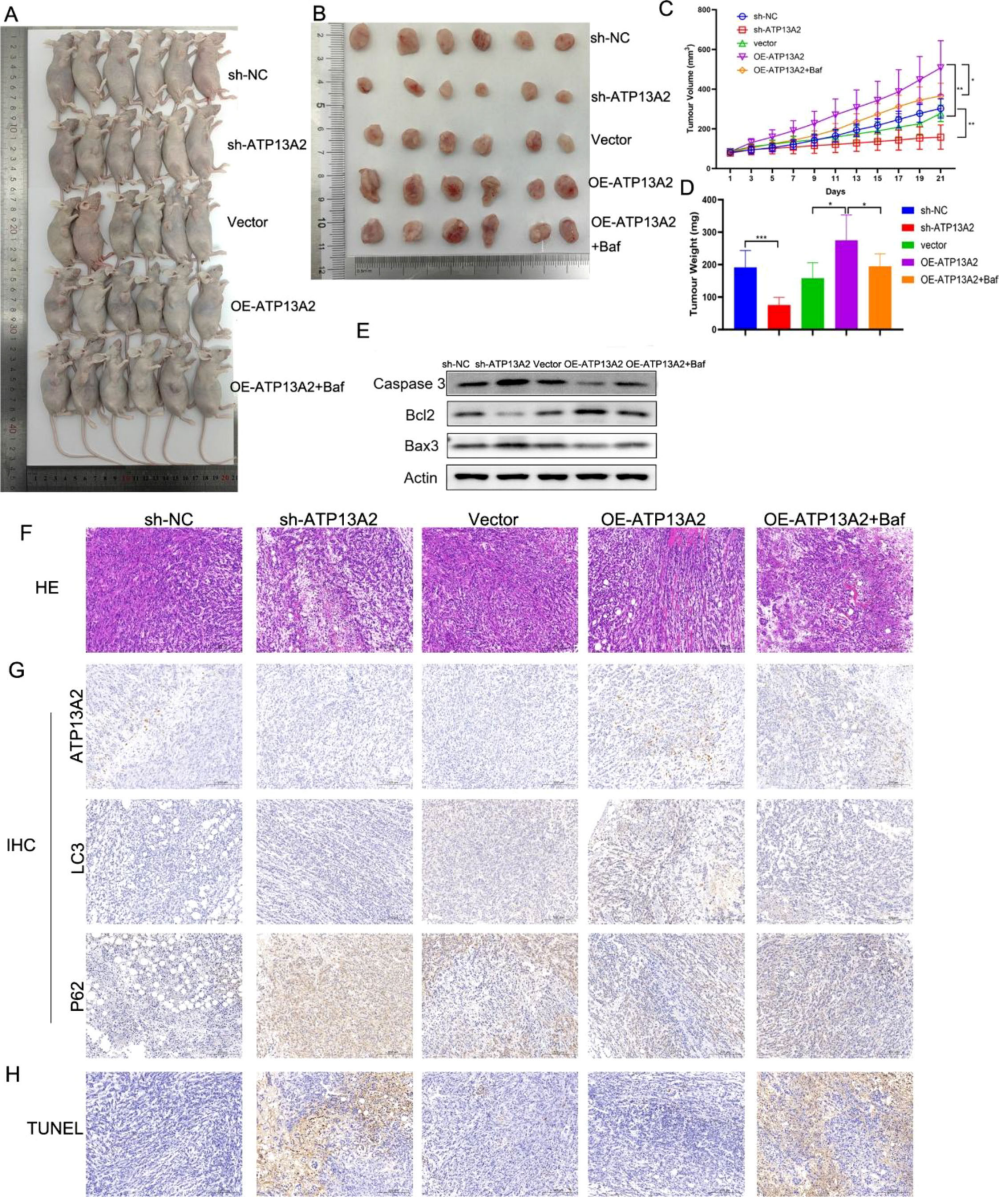
ATP13A2 promoted bladder cancer cell growth. **(A)** Mice were divided into sh-NC and sh-ATP13A2 groups (n = 6 per group). **(B-D)** The tumor size, volume, and weight were measured in mice treated with sh-NC or sh-ATP13A2. **(E)** The expression levels of apoptosis-related proteins (Caspase-3, Bcl-2, and Bax) were detected by western blot in tumor tissues. **(F)** H&E, Hematoxylin and eosin staining was performed to evaluate histopathological changes in tumor tissues. **(G)** IHC, Immunohistochemistry analysis was performed to assess the expression levels of ATP13A2, p62, and LC3 in tumor tissues. **(H)** Cell apoptosis was evaluated using TUNEL staining in tumor tissues. N = 6, data are presented as mean ± SD. Statistical significance was determined by one-way ANOVA followed by Tukey’s *post hoc* test. *P < 0.05. ***P < 0.001.

Tumor volume and weight were significantly reduced by sh-ATP13A2 and increased by OE-ATP13A2 (*P* < 0.001; **Figures 8A–D**). These results confirmed the role of ATP13A2 in promoting tumor cell growth. Compared with the OE-ATP13A2 group, the tumor size, volume, and weight in the OE-ATP13A2+Baf group were significantly decreased (*P* < 0.05, **Figures 8B–D**), demonstrating that autophagy inhibitor inhibited the tumor growth. The apoptosis biomarkers of Bax3 and Caspase3 were remarkably increased by sh-ATP13A2, and decreased by OE-ATP13A2. Opposed expression trend was observed in Bcl2. These results demonstrated that sh-ATP13A2 promoted cell apoptosis. Additionally, OE-ATP13A2+Baf showed anti-function with OE-ATP13A2 (**Figure 8E**). H&E staining showed that the cells were significantly inhibited by sh-ATP13A2 and promoted by OE-ATP13A2 (**Figure 8F**). IHC evaluated the expression levels of ATP13A2, LC3, and p62, and the results showed that p62 was highly expressed in the sh-ATP13A2 group when compared with the sh-NC group, and was lowly expressed in OE-ATP13A2 when compared with vector (**Figure 8G**). LC3 exhibited an opposite regulation trend with p62 in these groups. TUNEL staining showed that cell apoptosis was enhanced by sh-ATP13A2 (**Figure 8H**). To further validate the role of ATP13A2 in an immunocompetent context, we established an MB49 syngeneic bladder cancer mouse model. Compared with the sh-NC group, mice in the sh-ATP13A2 group exhibited significantly reduced tumor volume and weight, consistent with the xenograft results (**Supplementary Figures 7A–D**). IHC analysis further revealed markedly decreased PD-L1 expression and significantly increased CD8^+^ T cell infiltration in sh-ATP13A2 tumors, suggesting that ATP13A2 knockdown may alleviate tumor immune suppression and enhance anti-tumor immune responses (**Supplementary Figure 7E**). Taken together, these results indicate that ATP13A2 promotes bladder cancer cell growth, and bafilomycin A1 is an essential factor that slows tumor growth.

## Discussion

Growing evidence indicates that the stem-like properties of cancer cells contribute to tumor initiation, progression, and resistance to treatment (16). Huang et al. (14) have demonstrated that ATP13A2 facilitates tumorigenesis by regulating autophagic flux. Moreover, ATP13A2 is emerging as a new prognostic biomarker and a promising therapeutic target. As detected by GEPIA, ATP13A2 is an essential molecular component associated with the poor prognosis of bladder cancer. However, the molecular mechanism of ATP13A2 regulating bladder cancer progression is still unclear. In the present study, we first verified the role of ATP13A2 in regulating bladder cancer stemness, autophagy, and cisplatin resistance. Then, we explored the function of ATP13A2 in affecting bladder cancer cell progression. We can draw the following conclusions: (1) ATP13A2 is upregulated in bladder cancer; (2) Silencing ATP13A2 inhibits cell stemness, cisplatin resistance, autophagy, and cell progression; (3) the autophagy inhibitor bafilomycin A1 significantly inhibits cell stemness, weakens drug resistance, and suppresses cell progression; (4) knockdown of ATP13A2 inhibits bladder cancer cell growth; and (5) bafilomycin A1 plays an essential role in slowing tumor growth.

We found that ATP13A2 was upregulated in bladder cancer and associated with poor prognosis. The expression level of ATP13A2 varies across different cancers. For example, it is downregulated in brain cancer (13) and upregulated in hepatocellular carcinoma (HCC) (17), colorectal cancer (18), and colon cancer (14). Zheng et al. (19) have highlighted two types of nervous system tumors, glioblastoma multiforme (GBM) and lower-grade glioma (LGG), show low ATP13A2 expression, whereas most other cancers exhibit upregulated ATP13A2. Additionally, colon cancer patients with higher ATP13A2 levels tend to have shorter overall survival (14). Likewise, patients with HCC with higher ATP13A2 levels also experience poorer prognoses and promote cell progression (17). We analyzed the regulation of ATP13A2 in bladder cancer for the first time. Huang et al. (17) provided evidence that ATP13A2 may work as an oncogenic factor and a prognostic biomarker for HCC. ATP13A2 stimulates colorectal cancer growth by activating the pentose phosphate pathway via the TFEB-PGD axis (18). Therefore, the function of ATP13A2 in cancer varied, and more research was needed.

In several studies, ATP13A2 has been directly associated with mitophagy (20–22), with the mechanisms being little known. Besides its involvement in mitophagy, ATP13A2 has been linked to protein autophagy (23–25) and the regulation of metal and cation homeostasis (26–28). These functions are also critical to cellular processes related to alpha-synuclein biology. As we revealed, ATP13A2 regulates bladder cancer associated with autophagy, which is also demonstrated in colon cancer. As reported, the knockdown of ATP13A2 diminishes tumorigenesis by inhibiting autophagic flux in colon cancer (14). Cells with reduced ATP13A2 expression showed a decline in autophagic flux, corresponding to elevated levels of phospho-mTOR and a reduced response to autophagy induction by rapamycin (21). This decrease in autophagy linked to ATP13A2 deficiency impacts mitochondrial quality control, raising reactive oxygen species. ATP13A2 has also been implicated in autophagy pathways in Parkinson’s disease (29, 30). Little attention has been paid to the integration between ATP13A2 and autophagy in cancer, which may represent an important research direction. Notably, accumulating evidence suggests that dysregulated autophagy can profoundly affect tumor immunity by modulating antigen processing, immune checkpoint expression, and immune cell recruitment (31). In line with this concept, our immune infiltration analyses based on TCGA-BLCA data revealed that ATP13A2 expression was negatively correlated with StromalScore and ESTIMATEScore, indicating reduced immune and stromal components in tumors with high ATP13A2 expression. ssGSEA further demonstrated that ATP13A2 was negatively associated with antitumor immune populations, including CD8^+^ T cells, B cells, and plasmacytoid dendritic cells, while showing a positive correlation with Th2 cells and γδ T cells. These findings suggest that ATP13A2 overexpression may contribute to an immunosuppressive tumor immune microenvironment (TIME) characterized by impaired cytotoxic immune surveillance. To further validate these findings in an immunocompetent context, we established an MB49 syngeneic bladder cancer mouse model. Compared with control tumors, ATP13A2-silenced tumors exhibited significantly reduced tumor growth, accompanied by markedly decreased PD-L1 expression and increased CD8^+^ T cell infiltration. These data provide *in vivo* evidence that ATP13A2 contributes to tumor immune suppression and that its inhibition may restore antitumor immune responses. Collectively, these findings position ATP13A2 as a critical regulator linking autophagy, tumor progression, and immune evasion in bladder cancer.

From a clinical perspective, our findings suggest several potential implications for bladder cancer management. First, the strong association between high ATP13A2 expression and poor prognosis indicates that ATP13A2 could serve as a prognostic biomarker to identify patients at higher risk of disease progression and treatment failure. Second, given that ATP13A2 promotes cisplatin resistance, patients with high ATP13A2 expression may benefit from alternative or combination therapeutic strategies, such as autophagy inhibition or immune checkpoint blockade. Third, the successful suppression of tumor growth by bafilomycin A1 in our animal models raises the possibility that targeting ATP13A2 or its downstream autophagic pathways could be developed as a novel therapeutic approach, particularly for cisplatin-resistant bladder cancer. Fourth, the immunomodulatory role of ATP13A2, evidenced by PD-L1 regulation and CD8^+^ T cell infiltration, suggests that ATP13A2 inhibition may enhance the efficacy of existing immunotherapies, such as anti-PD-1/PD-L1 antibodies. Collectively, these clinical implications warrant further validation in prospective patient cohorts and preclinical models.

Bafilomycin A1 is a specific and potent inhibitor of H+-ATPase at the plasma membrane (32). Bafilomycin A1 can inhibit the growth of tumors by inhibiting the function of acidic glands (such as lysosomes) in tumor cells, affecting the metabolism and proliferation of tumor cells (33). Bychkova et al. (34) demonstrated that bafilomycin A1 may be applicable for cancer therapy, which is consistent with our present study. Moreover, bafilomycin A1 not only preferentially targets the leukemia stem cells derived from B-cell acute lymphoblastic leukemia patients but is also well-tolerated by normal primitive hematopoietic cells (35). Therefore, bafilomycin A1 could be explored as a promising therapeutic target for bladder cancer.

Chen et al. (14) demonstrated that ATP13A2 is linked to the stemness of colon cancer cells. Our present study showed that ATP13A2 regulates cisplatin resistance and stemness, which was reported for the first time in bladder cancer. Compared with previous studies in other cancer types, our work provides several novel insights. While Zhao et al. (36) and Huang et al. (17). focused primarily on the prognostic value and immune correlation of ATP13A2 in HCC and cervical cancer, respectively, the present study goes further by experimentally validating the functional role of ATP13A2 in regulating autophagy, chemoresistance, and stemness through both *in vitro* and *in vivo* models. The novelty of this study lies not only in identifying ATP13A2 as an oncogenic driver of bladder cancer progression and chemoresistance but also in uncovering its previously unappreciated role as an immune-modulatory molecule. By linking autophagy regulation to immune checkpoint expression and immune cell infiltration, our findings highlight the potential of ATP13A2 as a biomarker and therapeutic target for immunotherapy-based combination strategies in bladder cancer.

## Conclusion

We investigated the function of ATP13A2 in regulating bladder cancer in terms of autophagy, cisplatin resistance, stemness, and cell progression. We found that ATP13A2 was upregulated in bladder cancer and associated with poor prognosis. Additionally, the highly expressed ATP13A2 promoted cell stemness, cisplatin resistance, autophagy, and cell progression. Taken together, our study provides a novel biomarker for bladder cancer that may serve as a critical therapeutic target. Moreover, these findings expand the biological significance of ATP13A2 into the field of cancer immunity and immunotherapy, positioning ATP13A2 as a promising therapeutic target that integrates tumor-intrinsic oncogenic signaling with immune modulation in bladder cancer. Future studies should focus on validating the clinical utility of ATP13A2 as a prognostic biomarker in large patient cohorts, exploring the therapeutic efficacy of bafilomycin A1 or other autophagy inhibitors in combination with cisplatin or immune checkpoint inhibitors, and elucidating the detailed molecular mechanisms by which ATP13A2 regulates PD-L1 expression and immune cell recruitment.

## Data Availability

The original contributions presented in the study are included in the article/**Supplementary Material**, further inquiries can be directed to the corresponding author/s.
