# Supplementary material for: ATP13A2 promoted cell stemness, cisplatin resistance, autophagy, and cell progression of bladder cancer
Source: Front Immunol. 2026 May 7;17:1743064. doi: 10.3389/fimmu.2026.1743064 (PMC13189795; doi:10.3389/fimmu.2026.1743064)
Supplement: Supplementary file 1 [file DataSheet1.pdf]

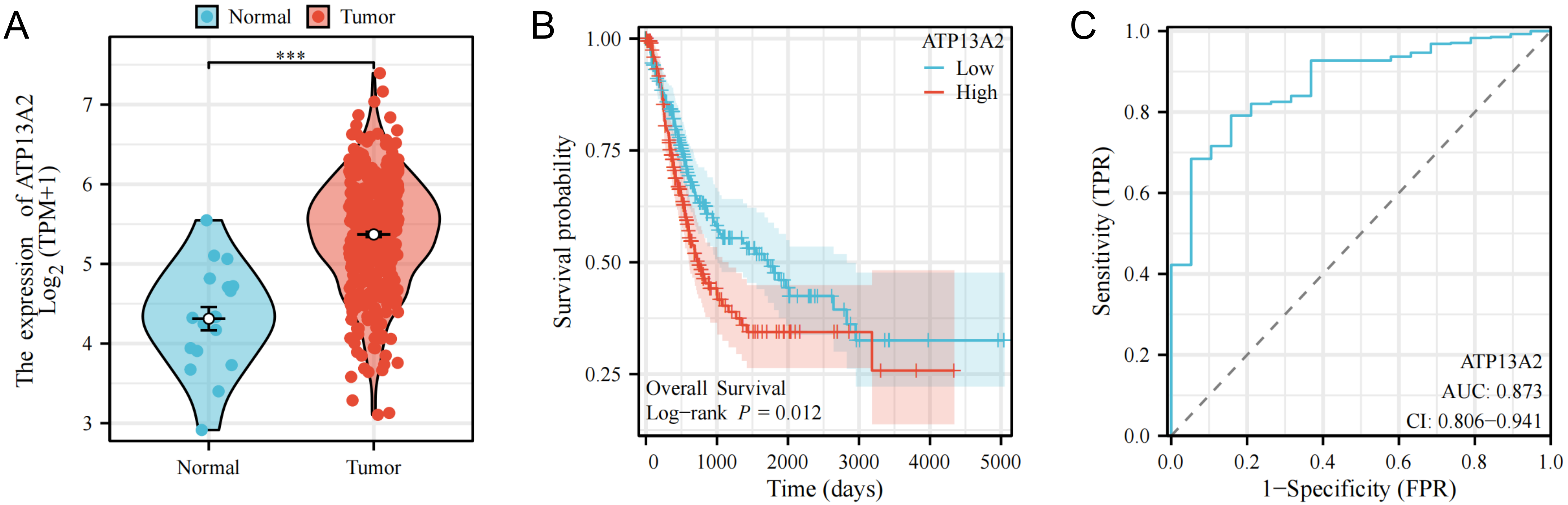

Supplement Figure 1. Clinical relevance of ATP13A2 expression in bladder cancer.
(A) Expression of ATP13A2 in bladder cancer tissues. (B) Survival analysis of ATP13A2. (C) ROC diagnostic curve analysis of ATP13A2. ***P<0.001.

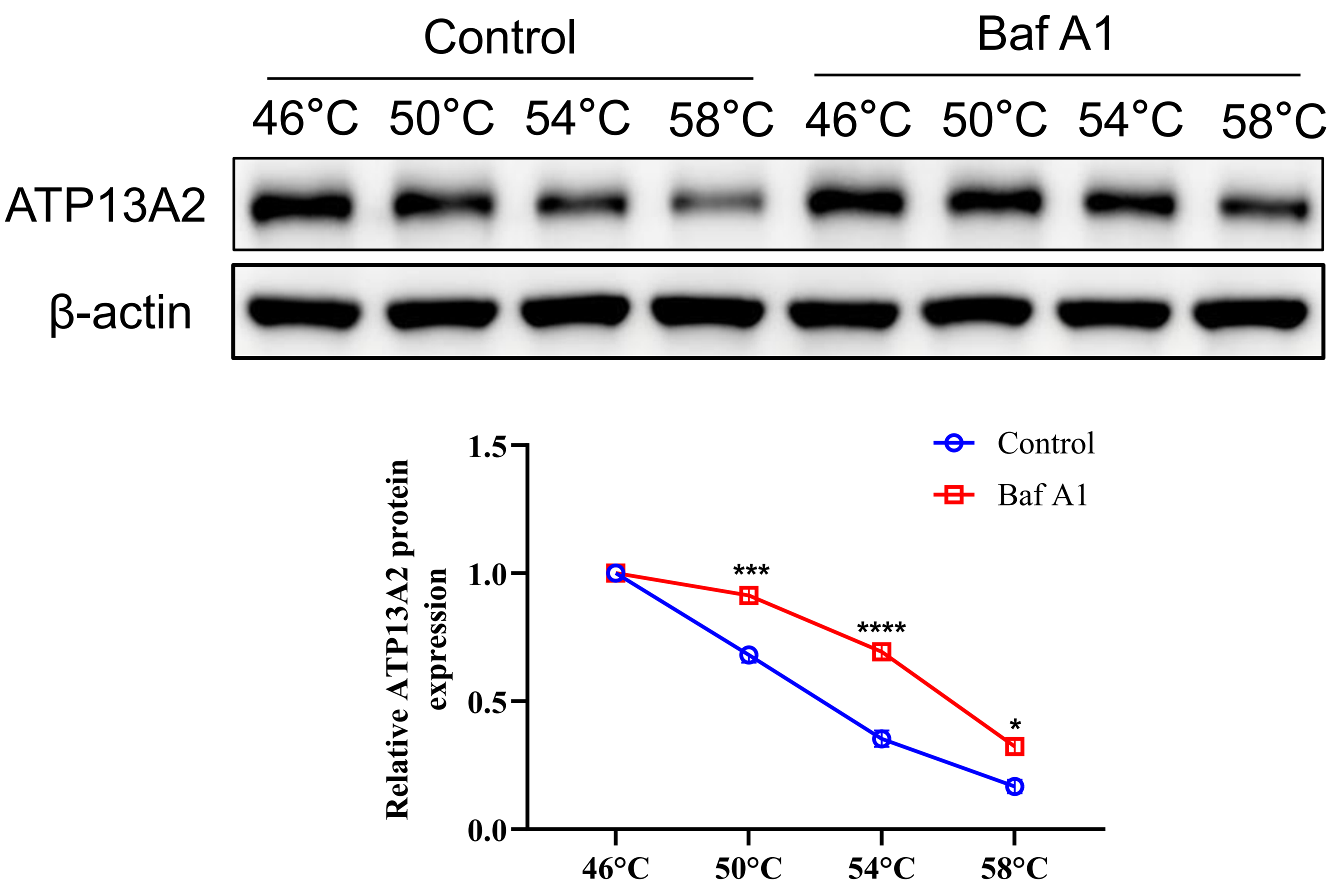

Supplement Figure 2. CETSA was performed to assess the direct intracellular interaction between bafilomycin A1 and ATP13A2. ***P<0.001, ****P<0.0001.

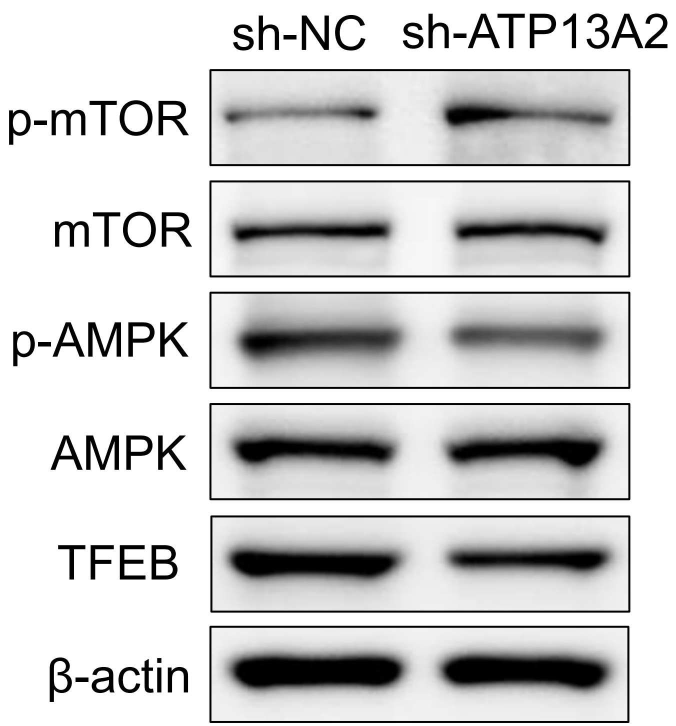

Supplement Figure 3. Western blot analysis of the ratios and expression levels of p-mTOR/mTOR, p-AMPK/AMPK, and TFEB in cells with ATP13A2 knockdown.

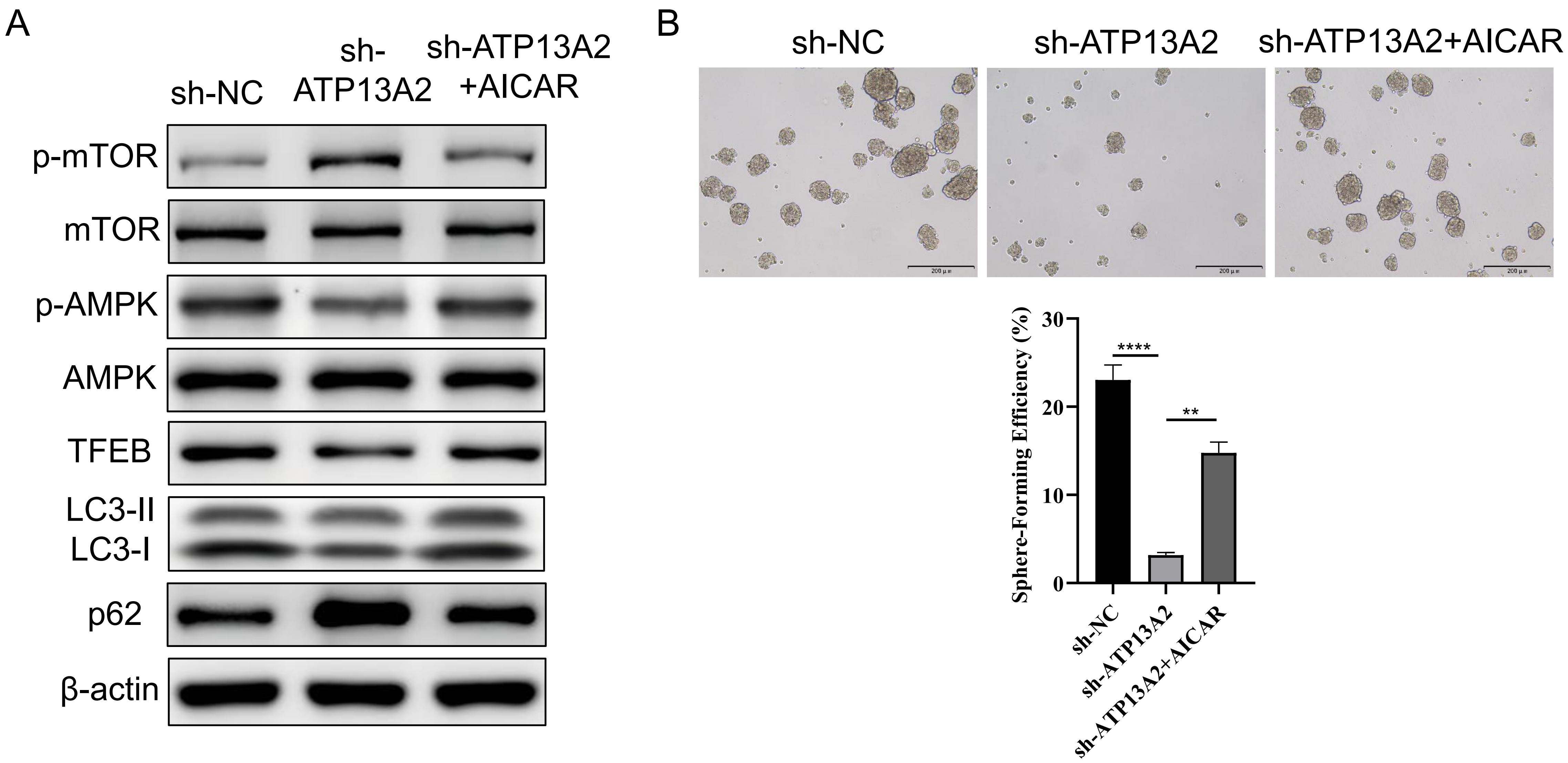

Supplement Figure 4. Effects of the AMPK activator AICAR on autophagy and cellular stemness. (A) Western blot analysis of AMPK/mTOR/TFEB signaling pathway and autophagy-related protein expression. (B) Cell sphere formation assay was performed to assess cellular stemness. **P<0.01, ****P<0.0001.

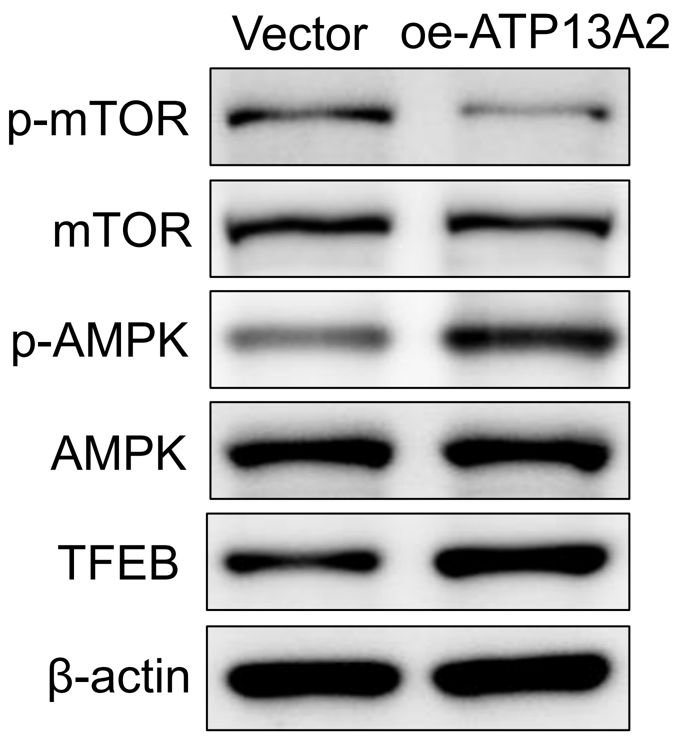

Supplement Figure 5. Western blot analysis of the ratios and expression levels of p-mTOR/mTOR, p-AMPK/AMPK, and TFEB in cells with ATP13A2 overexpression.

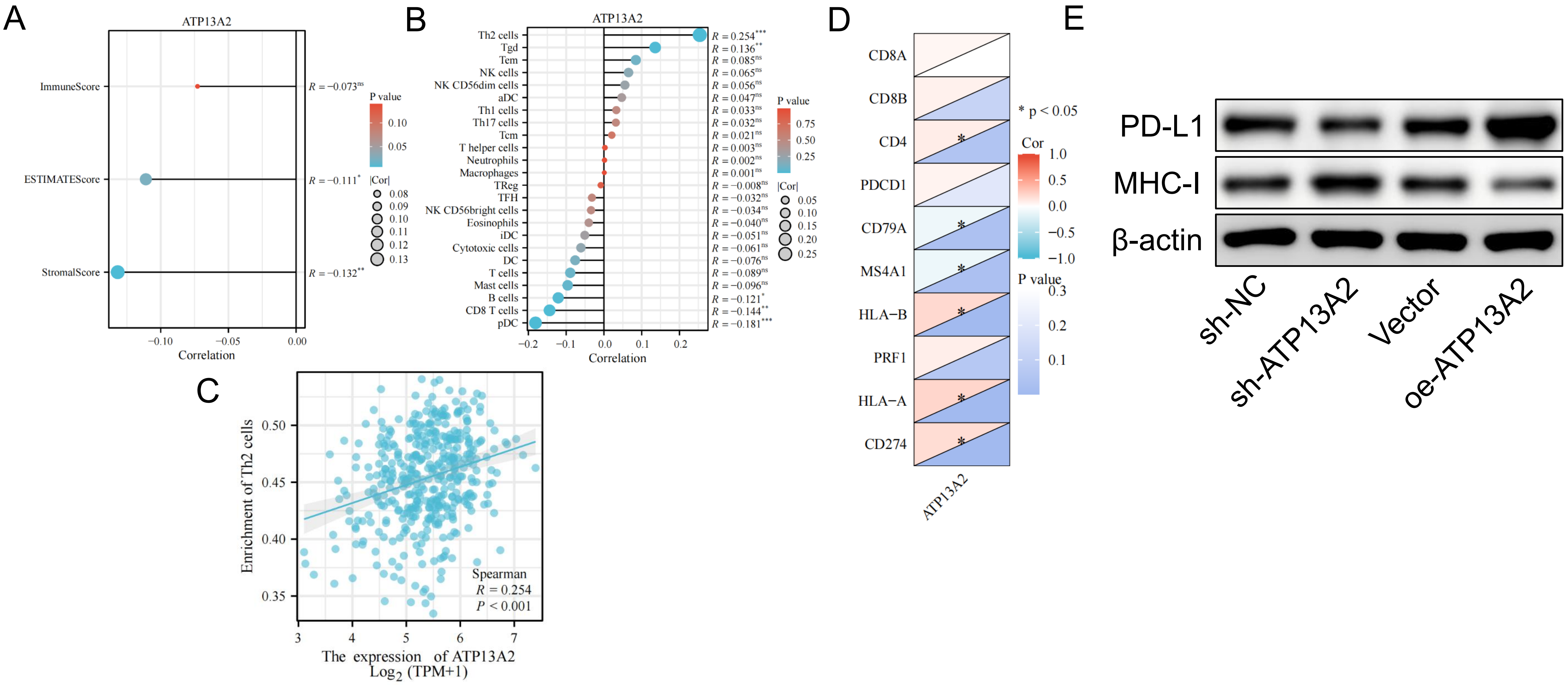

Supplement Figure 6. ATP13A2 is associated with an immunosuppressive tumor immune microenvironment. (A) The correlation between ATP13A2 and immune infiltration in bladder cancer was evaluated using the ESTIMATE algorithm. (B) The correlation between ATP13A2 and immune infiltration in bladder cancer was further assessed using the ssGSEA algorithm. (C) Scatter plot showing the correlation between ATP13A2 and immune infiltration in Th2 cells. (D) Heatmap depicting the correlation between ATP13A2 and immune-regulatory genes in bladder cancer. (E) The expression levels of immune-related proteins PD-L1 and MHC-I were assessed by Western blot analysis. *P<0.05, **P<0.01, ***P<0.001.

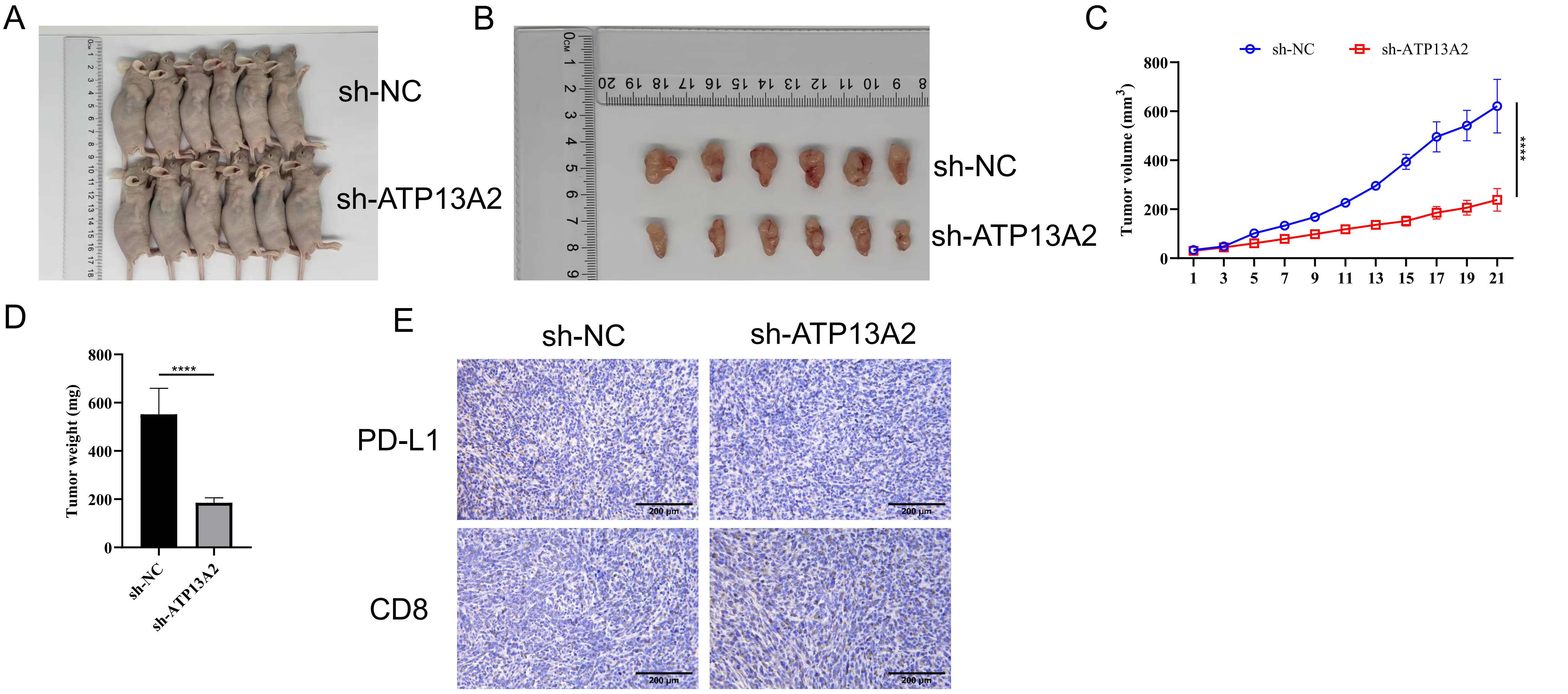

Supplement Figure 7. Effects of ATP13A2 knockdown on tumor growth and immune responses in the MB49 syngeneic bladder cancer mouse model. (A) The animals are in groups. (B-D) The tumor size, volume, and weight were measured under the treatments of sh-NC and sh-ATP13A2. (E) IHC analysis is used to evaluate PD-L1and CD8 expression levels in groups. ****P<0.0001.
